# Delayed gut microbiota development in high-risk for asthma infants is temporarily modifiable by *Lactobacillus* supplementation

**DOI:** 10.1038/s41467-018-03157-4

**Published:** 2018-02-16

**Authors:** Juliana Durack, Nikole E. Kimes, Din L. Lin, Marcus Rauch, Michelle McKean, Kathryn McCauley, Ariane R. Panzer, Jordan S. Mar, Michael D. Cabana, Susan V. Lynch

**Affiliations:** 1 Division of Gastroenterology, Department of Medicine, University of California San Francisco, San Francisco, CA 94143 USA; 2 Division of General Pediatrics, Department of Pediatrics, University of California San Francisco, San Francisco, CA 94143 USA; 3 Division of Clinical Epidemiology, Department of Epidemiology and Biostatistics, University of California San Francisco, San Francisco, CA 94143 USA; 4 Present Address: Siolta Therapeutics, 953 Indiana Street, San Francisco, CA 94107 USA; 5 Present Address: Janssen Prevention Center, 2 Royal College Street, London, NW1 0NH UK; 6 Present Address: Genentech, 340 Pt. San Bruno Boulevard, South San Francisco, CA 94080 USA

## Abstract

Gut microbiota dysbiosis and metabolic dysfunction in infancy precedes childhood atopy and asthma development. Here we examined gut microbiota maturation over the first year of life in infants at high risk for asthma (HR), and whether it is modifiable by early-life *Lactobacillus* supplementation. We performed a longitudinal comparison of stool samples collected from HR infants randomized to daily oral *Lactobacillus rhamnosus* GG (HRLGG) or placebo (HRP) for 6 months, and healthy (HC) infants. Meconium microbiota of HRP participants is distinct, follows a delayed developmental trajectory, and is primarily glycolytic and depleted of a range of anti-inflammatory lipids at 6 months of age. These deficits are partly rescued in HRLGG infants, but this effect was lost at 12 months of age, 6 months after cessation of supplementation. Thus we show that early-life gut microbial development is distinct, but plastic, in HR infants. Our findings offer a novel strategy for early-life preventative interventions.

## Abstract (web summary)

Gut microbial dysbiosis in infancy is associated with childhood atopy and the development of asthma. Here, the authors show that gut microbiota perturbation is evident in the very earliest stages of postnatal life, continues throughout infancy, and can be partially rescued by *Lactobacillus* supplementation in high-risk for asthma infants.

## Introduction

Atopy, the failure to downregulate pro-inflammatory responses to typically innocuous stimuli, is among the most common affliction in the western world^1^ and frequently precedes childhood asthma development^2,3^. Independent birth cohorts indicate that the depletion of specific bacterial genera from the infant gut microbiota at 1 or 3 months old is associated with increased risk of atopy, recurrent wheeze, or asthma development in childhood^4,5^. One month old infants at significantly increased relative-risk for subsequent development of atopy or asthma in childhood, exhibit a distinct gut microbiota and fecal metabolome, characteristically depleted of dihomo-γ-linoleate, a precursor for anti-inflammatory prostaglandins, and docosapentanoic acid, an anti-inflammatory ω-3 polyunsaturated fatty acid^5^. Moreover, sterile fecal water from high-risk neonates promoted CD4+ IL4+ expansion and reduced CD4+ CD25+ FoxP3+ cell frequency  ex vivo^5^, indicating that differences in infant gut microbiome composition and metabolites can induce immune dysfunction that precedes childhood atopy and asthma development. In mice, manipulation of the gut microbiome via a high-fiber dietary intervention (which increases concentrations of short chain fatty acids (SCFAs)), or oral *Lactobacillus* supplementation, promotes pro-resolving local and remote mucosal immunity, including induction of T-regulatory cell populations, and reprogramming of hematopoietic-derived immune cell precursor populations^6,7^. This occurs, at least in part, via microbial production, or induction, of metabolites, which shape host immune cell effector phenotypes^6–8^. Thus, mounting evidence implicates a developmental origin for childhood atopy and asthma involving gut microbiome perturbation and associated metabolic dysfunction in very early-life.

From an ecological perspective, founder species, those to first colonize a previously pristine environment, frequently dictate biome conditions and influence both the pace and subsequent pattern of species accumulation in the developing ecosystem^9^. Using this theoretical framework, we hypothesized that neonates at high risk for asthma (HR) exhibit meconium gut microbiota dysbiosis and a reduced rate of gut bacterial diversification over the first year of life. We also rationalized that early-life daily oral supplementation of HR infants with *Lactobacillus rhamnosus* GG (LGG), up to 6 months of age, would alter the microbiome development of high-risk infants, and promote both the bacterial taxa and metabolites necessary for the induction of immune tolerance.

In this study, we show that children at HR for asthma, exhibit a distinct pioneer meconium microbiota, delayed gut microbial diversification and are depleted for a range of anti-inflammatory fecal lipids in infancy. These deficits are partly rescued following LGG supplementation, and the products of LGG-supplemented infant gut microbiomes at 6 months of age, were found to increase the number of regulatory T cells ex vivo. This tolerogenic effect appears to be contingent upon sustained supplementation, since these effects were lost 6 months after cessation of supplementation. Our findings indicate that early-life gut microbiome perturbation and delayed development are associated with increased risk of childhood atopy and asthma. Moreover, they indicate nascent gut microbiome manipulation offers a feasible approach for immunomodulation in humans, and offer a much-needed framework for therapeutic development and future studies.

## Results

### HR infants exhibit delayed gut microbiota diversification

We took advantage of a randomly chosen subset of infants enrolled in the double-blind, placebo controlled trial of infant probiotic supplementation (TIPS) study^10^; designed to examine the effects of early-life LGG supplementation on childhood allergy and asthma development, in a HR population (HR; *n* = 25). A cohort of healthy infants at low risk for asthma with no family history of atopy were included as controls (HC; *n* = 29). Repeated stool samples were collected from all participants at standardized times (birth, 1, 3, 6, 9, and 12 months of age) and subjected to parallel 16S rRNA-based microbiota profiling and, in subset of 6 and 12 month samples, to LC/MS metabolomic analyses (Supplementary Figure 1). Questionnaires were administered throughout the study to capture demographic, disease, and dietary information.

Adherence to supplementation over the 6-month period was assessed by both quantitative PCR and sequence-based abundance of LGG which confirmed a significantly higher relative abundance of *Lactobacillus* in high-risk LGG-supplemented (HRLGG) compared to placebo (HRP) treated infants during the active supplementation period (Supplementary Figure 2a and b). Compared to HC subjects, HR infants were less likely be exposed to pets (Fisher’s exact test; *p* = 0.01; Supplementary Table 1), more likely to have a mother or father with either a history or active asthma (clinical trial inclusion criterion; Fisher’s exact test; *p* < 0.0001 for each), and trended toward increased rates of eczema at 12 months of age (Fisher’s exact test; *p* = 0.07). No significant differences in the proportion of subjects that were exclusively breast milk, formula or combination fed were observed between HC and HR subjects across the first year of life (*p* = 0.94). However, a cross-sectional analysis at each time point revealed that at the 12 month time point, HR infants were more likely to be exclusively breastfed and less likely to be combination fed or exclusively fed solid foods (Fisher’s exact test; *p* = 0.05; Supplementary Table 1). A comparison of HRP and HRLGG groups revealed no significant differences across any of the variables assessed (Supplementary Table 1).

Consistent with previous observations^5,11,12^ all participants exhibited progressive bacterial diversification and evidence of microbial succession over the first year of life as indicated by a positive relationship between age and bacterial alpha (Faith’s Phylogenetic diversity index; linear mixed effects (LME) model; *β* = 4.0, *p* < 0.0001; Supplementary Figure 3a) and beta diversity (unweighted UniFrac distance; LME model, *p* < 2 × 10^−16^; Supplementary Figure 3b). Of the factors examined (Supplementary Table 2), age, exclusive formula feeding and duration of breast feeding, in the first year of life were significantly associated with temporal variation in bacterial beta diversity (LME model; *p* < 0.0001, *p* < 0.0001, and *p* = 0.0005, respectively, Supplementary Figure 3b). These findings mirror observations from a previous infant gut microbiota study^11^. Mode of birth was not significantly associated with temporal variance in bacterial beta-diversity over the first year of life, which has been previously reported^13^ though this observation is likely underpowered (*n* = 4 Caesarian section delivered infants) in our study.

**Fig. 1:**
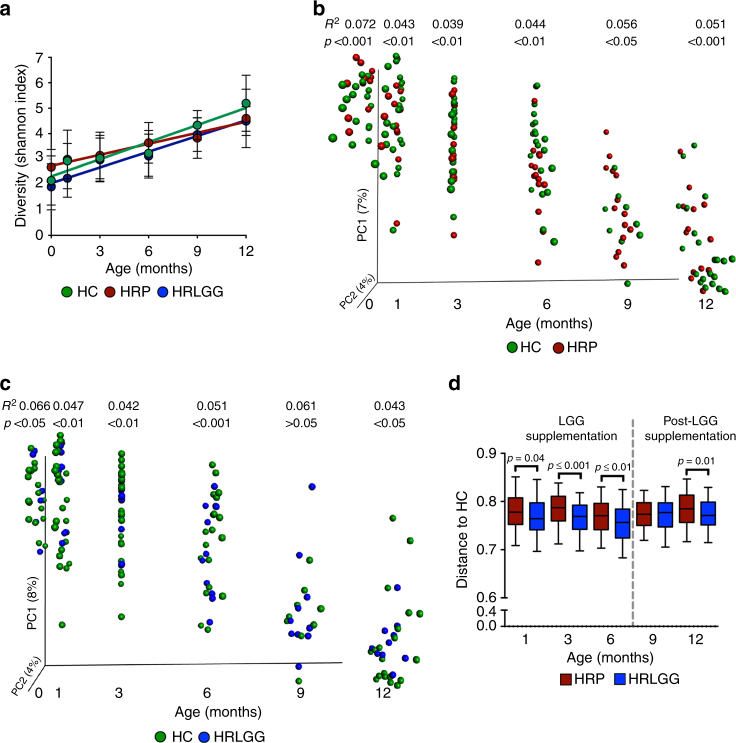
Gut microbiota maturation of high risk for asthma (HR) subjects is significantly distinct from that of healthy controls (HC), and influenced by oral *Lactobacillus rhamnosus* (LGG) supplementation. **a** Compared to HC (*n* = 29) subjects, HR placebo supplemented (HRP; *n* = 15) participants exhibit a significant delay in bacterial alpha diversification over the first year of life (*β* ANCOVA, *p* = 0.02), which is partly rescued by LGG supplementation (HRLGG; *n* = 10; *β *ANCOVA, *p* = 0.63). Error bars indicate standard-deviation from the mean. **b** HRP (*n* = 15) and **c** HRLGG (*n* = 10) infants exhibit significant differences in bacterial beta diversity across all of the time points assessed (Unweighted UniFrac distance; *R*^2^ and *p*-values calculated using PEMANOVA). **d** HRLGG (*n* = 10) infant gut microbiota is significantly (though marginally) more similar to HC (*n* = 29) subjects during the period of LGG supplementation (Unweighted UniFrac distance; Bonferroni corrected *t*-test). Whiskers extend to 95% confidence interval

Compared to their HC counterparts, HRP subjects demonstrated delayed gut microbiota diversification over the first year of life (Fig. 1a; LME model; *β* = 0.22 vs *β* = 0.14, respectively; *β* ANCOVA, *p* = 0.02), due to reduced rates of gain in both community richness and evenness (Supplementary Figure 4a and b). In contrast, HRLGG subjects exhibited a rate of bacterial gut microbiota diversification comparable to that of the HC group (Fig. 1a; LME model, *β* = 0.21, *β* ANCOVA, *p* = 0.63). However, LGG supplementation only rescued defects in community evenness; bacterial richness gains in the HRLGG group remained significantly lower than that of HC participants (Supplementary Figure 4a and b). These data indicate that while LGG supplementation influences taxonomic distributions within the developing HR infant gut microbiota, it fails to mitigate deficiencies in bacterial species accumulation which presumably are sourced from the infant’s local environment^11,14^. This observation is consistent with recent studies indicating that infants raised in residences with reduced house dust bacterial diversity are at heightened risk of developing atopy and recurrent wheeze in childhood^15–18^. A cross-sectional analysis of HC, HRP, and HRLGG at each time point, demonstrated that both HRLGG and HRP groups exhibit reduced richness at 12 months of age (Kruskal–Wallis, *p* = 0.02), irrespective of mode of nutrition (Linear model; adjusted change in estimate of 7%), implicating compounding gut microbiota dysbiosis with increased age in HR infants. Bacterial beta-diversity comparisons at each time point show significant and sustained differences in the composition of the gut microbiota of both HRP (Fig. 1b) and HRLGG (Fig. 1c) compared to HC infants throughout the first year of life. A between-group distance comparison indicates that during supplementation, HRLGG infants exhibit a gut microbiota composition that is marginally more similar to that of HC subjects (Fig. 1d). This suggests that LGG supplementation influences a relatively small subset of bacterial taxa in the developing gut microbiome of HR infants.

### Meconium microbiota are distinct in HR and HC infants

**Fig. 2:**
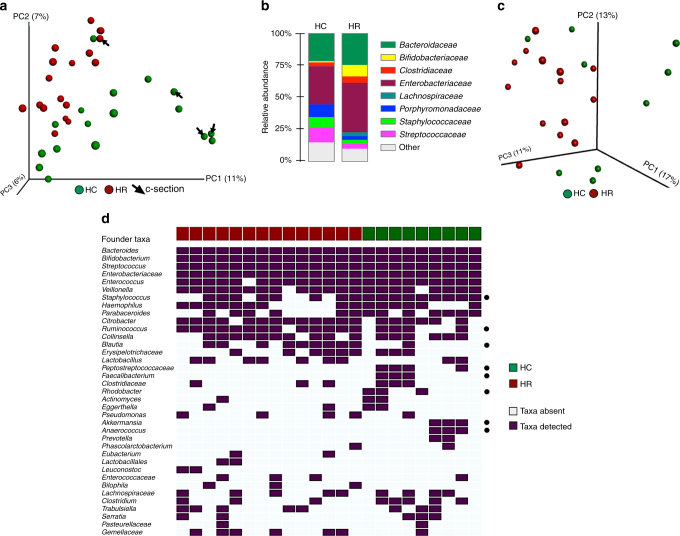
Meconium microbiota and persistent founder taxa significantly differ in high risk for asthma (HR) and healthy control (HC) subjects. **a** Principal Coordinates analysis (PCoA) of meconium microbiota indicates significantly different composition based on asthma risk status (HR *n* = 18; HC *n* = 17; Unweighted UniFrac; PERMANOVA *R*^2^ = 0.062, *p* = 0.001). **b** Taxon summary plot indicates expansion of *Enterobacteriaceae* and *Bacteroidaceae* in vaginally born HR (*n* = 17) meconium compared to HC (*n* = 14) neonates. **c** Persistent founder microbiota differ in HR (*n* = 14) and HC (*n* = 9) participants (Unweighted UniFrac; PERMANOVA *R*^2^ = 0.081, *p* = 0.018). **d** While a number of persistent founder taxa are common to both HC (*n* = 9) and HR (*n* = 14) subjects, several differ (Fisher’s exact test; *p* < 0.05; black dots), including a number whose depletion has previously been associated with atopy and asthma development in childhood

The greatest degree of variation in HR and HC community composition was observed in meconium samples (Fig. 2a); this was true whether C-section delivered infants were included or excluded from this analysis (PERMANOVA *R*^2^ = 0.062, *p* < 0.001 and *R*^2^ = 0.060, *p* < 0.001, respectively; Supplementary Table 3). Of the factors examined, parental asthma and active parental disease explained the greatest degree of meconium taxonomic variability (Supplementary Table 3), implicating both maternal and paternal health status as influential in early-life gut microbiome development. As previously described in refs. ^13,19,20^, C-section delivery also explained a proportion of the observed variance in meconium microbiota composition (*R*^2^ = 0.053, *p*-value = 0.001), though it should be noted that only a single-C-section delivered neonate clustered with HR participants in our study (Fig. 2a). Compared to HC, HR meconium was enriched for *Enterobacteriaceae* and *Bacteroidaceae*, and depleted of multiple genera, including *Akkermansia*, *Faecalibacterium*, and *Rothia* (Fig. 2b and Supplementary Data 1), the latter representing genera previously described as depleted from the feces of older infants (1 and 3 months old) at heightened risk of atopy, recurrent wheeze, and asthma later in childhood^4,5^. Thus, bacterial depletions characteristic of infants at increased risk for childhood atopy and asthma, are evident in the first postnatal stool of HR infants.

We hypothesized that despite the dynamics of gut microbiota succession, a small group of founder bacterial taxa, present in meconium, persist throughout the first year of gut microbiome development and that these taxa differ between HC and HR infants. The median number of persistent founder taxa (defined as taxa detected in meconium and all subsequent samples collected at 1, 3, 6, and 12 months) was small and did not differ between HC (*n* = 9) and HR (*n* = 14) groups (Median/IQ range HC: 66/47-91; HR: 64/57-83; Mann–Whitney, *p* = 0.9), though the composition of these persistent founder communities was distinct in HC and HR participants (Unweighted UniFrac; PERMANOVA, *R*^2^ = 0.081, *p* = 0.018; Fig. 2c). While specific persistent founder taxa within the *Enterobacteriaceae, Streptococcus, Bifidobacterium*, and *Bacteroides* were common to all infants (Fig. 2d), important taxonomic differences were observed between HR and HC subject persistent founder populations; HR infants were more likely to be persistently colonized by specific taxa within the *Blautia* and *Ruminococcus*, while HC subjects maintained specific *Peptostreptococcaceae, Staphylococcus, Anaerococcus, Rhodobacter, Akkermansia*, or *Faecalibacterium* members in their gut microbiota throughout the first year of life (Fisher’s exact test, *p* < 0.05; Fig. 2d). It is notable that several of these persistent founder taxa observed in HC subjects were, in independent cross-sectional studies, among those genera found to be significantly depleted from 1 to 3-month-old gut microbiota of infants at heightened risk of developing atopy, recurrent wheeze, and asthma in childhood^4,5^. More specifically key members of these genera are known to play important roles in pH modulation^21,22^, production of SCFAs, and influence mucin production and metabolism^23,24^, factors that strongly influence the colonization landscape for the developing gut microbiome. Hence, sustained presence and activities of specific founder microbes may represent important determinants of microbial developmental trajectories in early-life.

### LGG supplementation enriches for specific fecal taxa and metabolites

An assessment of temporal taxonomic enrichments in HC versus HRP subjects over the first year of life identified other specific taxa within notable genera including *Bifidobacteria*,* Lactobacillus*,* Ruminococcus*, *Clostridium*, *Bacteroides*, and *Blautia* that discriminated these infant groups, along an age-based developmental gradient (Supplementary Figure 5, Supplementary Data 2–5). Compared to HC subjects, HRP infants exhibited a notable and premature expansion of *Bacteroidaceae* and *Lachnospiraceae* at 3 months of age. Expansion of these families occurred much later in HC subjects (at 12 months), as has been described in previous studies^25^. By 12 months, relatively few bacterial taxa were significantly enriched in the HRP subjects, plausibly due our small sample size and bacterial community heterogeneity at this stage of development, or to expansion of non-bacterial species in the HRP gut microbiota^5^.

**Fig. 3:**
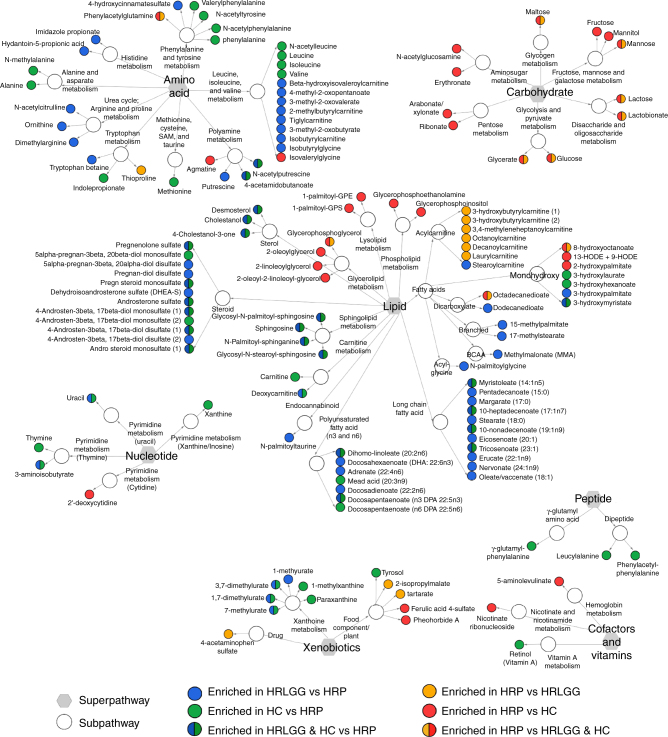
Three-way comparison of 6 month fecal samples identifies metabolites enriched in healthy controls (HC) and *Lactobacillus rhamnosus*-supplemented high risk for asthma (HRLGG) subjects compared to high risk for asthma placebo (HRP) group. HC (*n* = 15) and HRLGG (*n* = 7) participants share a number of common enriched metabolites compared with HRP (*n* = 11) group, including anti-inflammatory androgenic steroids, long-chain and polyunsaturated fatty acids. In comparison HRP subjects exhibit evidence of increased glycolysis and for distinct products of lipid metabolism (Welch’s two-sample *t*-test; *p* ≤ 0.05)

During and after the supplementation period, compared with HRP, HRLGG microbiota shared a greater degree of taxonomic overlap with HC subjects (Supplementary Figure 5 and 6a, Supplementary Data 6–9), though overall this represented a small proportion of the total taxa detected. To assess whether these alterations influenced gut microbiome function, un-targeted metabolomic analyses on a subset of 6 and 12-month-old paired fecal samples (HC *n* = 15; HRLGG *n* = 7; HRP *n* = 11) was performed. At 6 months of age, compared with HRP infants, microbial metabolism within the HRLGG gut was more similar to that of HC infants (Bray Curtis, Welch’s correction *t*-test, *p* < 0.0001; Supplementary Figure 6b and c). More specifically, compared to HRP infants, both the HC and HRLGG infants were characterized by enrichment of a diversity of lipid androgenic steroids (Fig. 3) known to be depleted in cases of chronic inflammation and shown to provide protection against various inflammatory conditions^26,27^. Fatty acids, of particular interest due to their anti-inflammatory roles in asthma and atopy^4,5,7,28^, represented some of the most highly enriched metabolites in both HC and HRLGG infant stool samples compared to that of HRP (Fig. 3), these included the linoleic acid derived omega-3 polyunsaturated fatty acids (ω-3 PUFA), docosapentaenoate (DPA; 22:5n3) and docosahexaenoate (DHA; 22:6n3), as well as dihomo-linoleate (20:2n6) and eicosatrienoic acid (ETA or mead acid; 20:3n9). Although the untargeted LC/MS approach was not optimized to quantify levels of SCFAs^7,29,30^, we noted an enrichment of 4-acetamidobutanoate, a precursor for alternative microbial SCFA biosynthesis^31^ in HC and HRLGG compared with HRP infants. In contrast, HRP infants exhibited evidence of inflammatory conditions in the gut, including significant enrichment of thioproline, which stimulates macrophage phagocytosis and chemotaxis toward inflammatory foci^32^ and of 9, 13 HODE, a pro-inflammatory metabolite of linoleic acid which can arise via P450 cytochrome activity or non-enzymatically, under oxidative conditions^33,34^ and is associated with tissue and DNA damage and, more recently, with severe asthma^35^. HRP infants also evidenced a pronounced shift towards predominantly glycolytic metabolism, as indicated by significant relative enrichment of a large range of simple sugars.

Contrasting the observations made in 6 months old samples (at the end of the supplementation period), 12 months old fecal metabolomes of the HRLGG group were no longer more similar to HC compared with HRP subjects (Supplementary Figure 6b and c). Only a small number of metabolites discriminated the three groups of infants at 12 months of age (Supplementary Figure 6b and 7) and HRLGG only exhibited the anti-inflammatory PUFA, DPA^36^, and microbial-derived odd-chain fatty acids 10-nonadecenoate and 10-hepatadecenoate^37^ (that possess T-cell stimulatory capacity^38^) in common with HC infants. We noted that compared to HRLGG, HRP infants were enriched for the tricarboxylic acid (TCA) cycle intermediate aconitate, a build-up of which is associated with inadequate bacterial clearance by macrophages^39^.

**Fig. 4:**
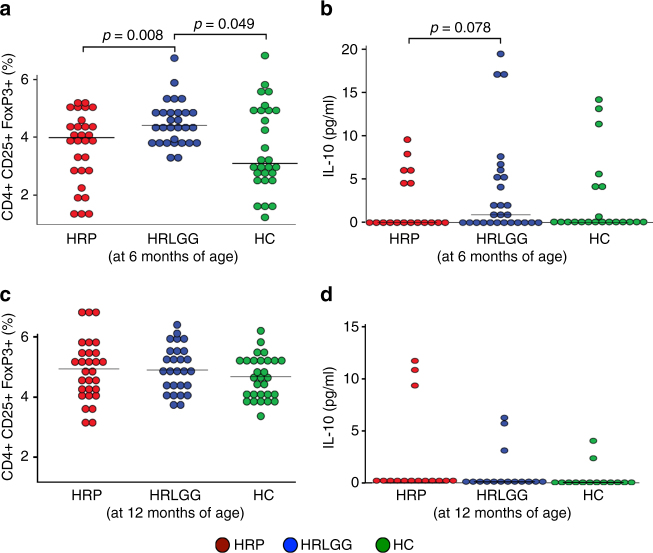
*Lactobacillus rhamnosus* (LGG)-associated fecal products promote *T*_reg_ cell expansion and IL10 production ex vivo at 6 months of age. Sterile fecal water derived from 6-month-old LGG-supplemented high risk for asthma (HRLGG; *n* = 5) stool induced an increase in the proportion of **a**. *T*_reg_ cells and **b**. IL10, compared with high risk for asthma placebo (HRP; *n* = 5) or healthy (HC; *n* = 5) participants. At 12 months of age, 6 months following cessation of LGG supplementation, no difference in the proportion of **c**. *T*_reg_ cells or **d**. IL10 expression was observed between the three groups. Linear mixed effects (LME) adjusted for blood-donor was used to test for between group differences

Based on our metabolic profiling data, we hypothesized that the gut microbiota and associated metabolic products in 6-month-old HRLGG infants promote tolerogenic conditions characterized by *T*_reg_ cell expansion. To test this hypothesis, we used a previously described ex vivo dendritic cell (DC)/T-cell assay^5^, and filter-sterile fecal water from a subset of samples (*n* = 5) from each group of infants. Compared to fecal water from 6-month-old HRP subjects, HRLGG induced significantly increased proportions of *T*_reg_ cells (LME model, *p* = 0.008, Fig. 4a) and the concentration of IL10 trended higher (LME model, *p* = 0.078, Fig. 4b). Fecal water derived from 12 months old samples produced no significant differences in *T*_reg_ populations across the three groups (Fig. 4c, d).

## Discussion

Independent cross-sectional studies have demonstrated with relative consistency, that the loss-of-specific bacteria in 1 or 3 months old infant gut microbiota is associated with increased risk for subsequent development of atopy, recurrent wheeze, or asthma in childhood^4,5^. Here we demonstrate that gut microbiota dysbiosis is evident in the first bowel movement of HR neonates and strongly co-associates with parental disease. Our observation mirrors a study in which maternal glycemic control, a proxy for diabetes, covaried with meconium bacterial microbiome^40^. These data indicate that parental health status during gestation may influence vertically inherited microbial populations that initiate gut microbiome development and influence immune maturation in early-life. Indeed, we provide the first evidence that HR infants exhibit a distinct population of founder bacterial taxa within meconium that persist over the first year of life, and a parallel compounding delay in bacterial species accumulation during the critical period of immune maturation, irrespective of LGG supplementation or mode of nutrition. The data presented support the hypothesis that the human gut microbiota adheres to the central tenets of ecological succession, and that differences in the composition of meconium founder populations are related to distinct gut microbiota developmental trajectories. The observation that a small number of specific bacterial taxa persist over the first year of life in the infant gut microbiome, may plausibly explain why very early-life microbial exposures are related to disease development in childhood. It is plausible that these primal species colonizing the neonatal and infant gut, influence subsequent microbiome development through persistent competitive colonization, resource sequestration, modulation of host immunity, or by influencing physiological development during this critical developmental window.

Of interest, while LGG supplementation rescued the microbiota evenness deficit observed in HRP infants, it did not mitigate the delay in bacterial species accumulation observed in these infants. HR infants in our study were significantly less likely to be exposed to pets which are known to increase residential house dust bacterial diversity^15–18^, implicating the built environment as a source of exogenous microbial species for the developing infant microbiome. Hence while LGG supplementation may partially rescue endogenous disparities in gut microbial distributions, it appears unable to compensate for reduced exposure to environmental microbes that may expand the diversity and functional repertoire of the developing gut microbiome.

Following 6 months of *Lactobacillus* supplementation, HRLGG subjects possessed a fecal metabolic milieu comprised of anti-inflammatory fatty acids known to promote immune tolerance in early infancy^41^. These observations are consistent with those made in an independent birth cohort of 1 month old infants at significantly lower risk of childhood allergic sensitization and asthma^5^. In both cases the gut microbiome associated products induced T-regulatory cells ex vivo, indicating that at least during the period of supplementation, LGG reprograms the composition and metabolism of the gut microbiome toward that which induces tolerogenic immunity, necessary to prevent atopy and asthma. However, the metabolic profile observed in HRLGG infants at 6 months was largely unsustained at 12 months of age and paralleled diminished LGG levels following cessation of supplementation. LGG does not encode the capacity to produce many of the anti-inflammatory lipids enriched in supplemented infants, instead, it promotes enrichment of fatty acid conjugating organisms, such as *Bifidobacteria*^42,43^, capable of their production. Thus, improved rationally designed microbial polybiotics comprising synergistic microbial species that successfully engraft and encode a broader range of functions including gene pathways for biosynthesis and metabolism of anti-inflammatory fatty acids, may be necessary to maintain durable effects on immune function in HR subjects. Alternatively, introduction of such microbes during pregnancy may promote their vertical transmission and early-life engraftment, offering a surrogate strategy for disease prevention, although much more work is needed to test the validity and safety of such an intervention.

Our study provides the first evidence that HR infants exhibit a distinct meconium bacterial community, microbiota maturation trajectory and fecal metabolome over the first year of life. It also indicates that daily supplementation with LGG leads to subtle but important taxonomic and metabolic remodeling in the nascent gut microbiota that promotes induction of T-regulatory populations ex vivo, but that these effects appear to be contingent upon sustained supplementation. Providing evidence for the malleability of the nascent gut microbiome, identifying the early postnatal period as a critical window for safe intervention, and ascertaining the functional features of a protective nascent gut microbiome offer a much-needed framework for novel interventions aimed at disease prevention.

## Methods

### Ethics statement

The Committee on Human Research at University of California San Francisco approved all study protocols, and all parents provided written, informed consent.

### Study outline and sample collection

Newborns at HR for asthma, born to at least one biological parent with asthma and enrolled in the TIPS study^10^ were randomized in blocks of four to daily (LGG^44^; strain ATCC 53103; at 1 × 10^10^ CFU; *n* = 10) or placebo (*n* = 15) supplementation for the first 6 months of life. Stool samples from 25 randomly selected participants enrolled in the TIPS study and 29 healthy (HC) participants enrolled in the Development of Infant Microbial Evolution study (DIMES), born to non-atopic parents, were collected at the following time points: 0 (first bowel movement), 1, 3, 6, 9, and 12 months. Only a small subset of HC infants had samples collected at 9 months, consequently this time point was excluded in a number of analyses. Samples were collected from diapers using a scoop attached to the lid of a sterile collection tube (Sarstedt, Germany), mailed overnight to the study team, and immediately banked at −80 °C upon receipt. Parents or caretakers were queried about clinical outcomes and feeding practices each month over the first year of life using standardized questionnaires.

### Fecal nucleic acid extraction

Infant stool samples were maintained at −80 °C until processing. Total DNA was extracted using the QIAamp DNA Stool Mini kit (Qiagen, CA) with slight modifications. Briefly, frozen stool samples were emulsified in 700 μl ASL buffer and transferred into Lysing Matrix E tubes (LME; MP Biomedicals, CA). Samples were homogenized in a FastPrep-24 instrument (MP Biomedicals, CA) at 6.0 m/s for 30 s. Beads were pelleted and the supernatant was transferred into sterile 2 ml microcentrifuge tube. Fresh ASL buffer (900 μl) was added to each LME tube, followed by repeated homogenization at 6.0 m/s for 30 s. Beads were pelleted and the supernatant combined with the supernatant from the first extraction step. DNA isolation was completed as outlined in the QIAamp DNA Stool Mini kit manual. DNA was eluted in 150 μl dH_2_O and stored at −20 °C.

### Quantitative PCR to assess abundance of LGG

To assess the presence and relative abundance of LGG, quantitative polymerase chain reaction (Q-PCR) was performed as previously described using LcF (5′-CGCATGGTTCTTGGCTGAAA-3′) and LcR (5′-ACAACAGTTACTCTGCCGAC-3′) primer pair^45^. A total of 20 ng of DNA per reaction was used in triplicate, 25 μl Q-PCR reactions at an annealing temperature of 55 °C. Samples with low total DNA yield (15 total) were assayed with DNA concentrations below 20 ng.

### PCR conditions and library preparation for sequencing

The variable region 4 (V4) of the 16S *rRNA* gene was amplified using 10 ng μl^−^^1^ of DNA template and 515F/806R primer combination as previously described^46^. Amplicons were purified using AMPure SPRI beads (Beckman Coulter), quality checked using Bioanalyzer DNA 1000 Kit (Aligent), quantified using the Qubit 2.0 Fluorometer and the dsDNA HS Assay Kit (Life Technologies) and pooled at 50 ng per sample. The denatured libraries were diluted to 2 nM, and 5 pM were loaded onto the Illumina MiSeq cartridge (V3) in combination with a 15% (v/v) of denatured 12.5 pM PhiX spike-in control as previously described^46^.

### Sequence data processing and quality control

Paired-end sequences were combined using FLASh version 1.2.7^47^. Sequences were processed using the Quantitative Insights into Microbial Ecology (QIIME) pipeline version 1.8.0^48^. Raw sequences were de-multiplexed by barcode and quality filtered by removing low-quality sequences. Sequences with three or more consecutive bases with a *Q*-score <30 were truncated and discarded if the length was <75% of the original 250 bp read length. Sequences were aligned using PyNAST^49^, chimera checked using usearch61^50^ and operational taxonomic units (OTUs) were picked at 97% sequence identity using UCLUST^50^ against the Greengenes database^51^ (version 13_5). Reads that failed to hit the reference sequence collection were retained and clustered de novo. A phylogenetic tree was built using FastTree^52^. To normalize variation in read-depth across samples, data were multiply rarefied to 218,687 sequences per sample as previously described^5^.

### Untargeted metabolomics analysis of infant stool samples

Paired stool samples (200 mg) collected at 6 months and 12 months (*n* = 33 pairs) from a subset of infants (HC *n* = 15, HRLGG *n* = 7, and HRP *n* = 11) at both 6 and 12 months were used for ultrahigh performance liquid chromatography/tandem mass spectrometry (UPLC–MS/MS) and gas chromatography–mass spectrometry (GC–MS) by Metabolon (Durham, NC), using their standard protocol (http://www.metabolon.com/). Compounds were compared to Metabolon’s in-house library of purified standards, which includes more than 3,300 commercially available compounds.

### Ex vivo dendritic cell challenge and T-cell coculture

Fecal water from a subset of paired samples (*n* = 15 pairs) with sufficient remaining material, that had previously undergone metabolic profiling (HC *n* = 5, HRLGG *n* = 5, and HRP *n* = 5) was resuspended (1 g/ml w:v) in phosphate-buffered saline (PBS) containing 20% fetal bovine serum (FBS) and filter-sterilized through a 0.2-μm filter as previously described^5^. Fecal water from each sample was used in the dendritic cell (DC)/T-cell assay described below.

Peripheral blood mononuclear cells (PBMCs) were isolated from two healthy human donors (Blood Centers of the Pacific, San Francisco, CA) using Ficoll–Hypaque gradient centrifugation, washed twice with R10 media (10% FBS, 2 mM l-glutamine and 100 U/ml penicillin-streptomycin). DCs were isolated from PBMCs using the EasySepTM Human Pan-DC Pre-Enrichment Kit (STEMCELL Technologies, Vancouver, BC). DCs (0.5 × 10^6^ cells/ml) from two donors and were treated at 25% v-v with either sterile fecal water or PBS (negative control) in triplicate and cultured in R10 media supplemented with 10 ng/ml granulocyte-macrophage colony-stimulating factor (GM-CSF) and 20 ng/ml IL4 at 37 °C. After 24 h of exposure, DC maturation was stimulated with growth mediators (10 ng/ml tumor nuclear factor alpha [TNF-α], 10 ng/ml IL-1β, 10 ng/ml IL-6, and 1 mM prostaglandin E2 [PGE2]), for an additional 24 h.

In preparation for coculture, DCs were washed in fresh R10 media, enumerated via flow cytometry and plated in TexMACs medium (Miltenyi Biotec, San Diego, CA) at 0.05 × 10^6^ live CD45+ cells per well. Autologous T lymphocytes were purified from the PBMCs using a naive T-cell isolation kit (Miltenyi Biotec), suspended in TexMACS medium and added to the treated DCs at a ratio of 10:1 in the presence of soluble anti-CD28 and anti-CD49d at 1 μg/ml (BD Biosciences, San Jose, CA). DCs and T cells were cocultured for 5 d at 37 °C and replenished with fresh TexMACS medium every 48 h.

The concentration of IL-10 cytokine in co-culture cell-free media, was measured using a cytometric bead array according to the manufacturer’s instructions (BD Bioscience, San Diego, CA) at the end of the DC/T cell co-culture experiment. To phenotype T-cell subsets via flow cytometry co-cultures were mixed with Phorbol Myristate Acetate-Ionomycin (Sigma, St. Louis, MO) and GolgiPlug (BD Biosciences) for 16 h. For flow cytometry, single-cell suspensions were stained using a panel of antibodies, including anti-CD3 (SP34-2), anti-CD4 (L200), anti-CD25 (M-A251), anti-IFNg (B27); anti-CD8a (RPA-T8); anti-IL4 (7A3-3); anti-IL-17A (eBio64DEC17), and anti-FoxP3 (PCH101). Dead cells were stained positive with LIVE/DEADAqua Dead Cell Stain (Life Technologies, Grand Island, NY). Permeabilization buffer (Affymetrix eBioscience) was used to permeabilize cell prior to staining for intracellular markers, IFNg, IL4, IL17A, and FoxP3. Flow cytometric data were acquired using the Flow Cytometer LSRII (BD Biosciences). For flow analysis, live T cells were gated as CD3+ CD4+ cells and T-regulatory cells were defined as CD4+, CD25^hi^, and FoxP3^hi^.

### Statistical analysis

When examining the association between early-life factors and the three study groups (HC, HRLGG, and HRP), or determining the difference in *Lactobacillus* relative abundance between HR groups, statistical significance was determined on the basis of covariate distribution by Kruskal–Wallis or Mann–Whitney *t*-test (numerical, skewed), or Fisher’s exact (sparse categorical).

Bacterial alpha diversity indices were calculated using Quantitative Insights Into Microbial Ecology (QIIME) for a subset of infants for which more than three samples were profiled in the study, and *β* ANCOVA (GraphPad Prism) was used to compare slopes of alpha diversity gain over the first year of life. Unweighted UniFrac^53^ distance was calculated in QIIME to assess compositional dissimilarity between samples, and visualized using PCoA plots constructed in Emperor^54^. Either alpha diversity indices or the first principal component coordinate (unweighted UniFrac) were used as a response variable in the LME model^55^ (lmerTest package in R) to determine the relationship between observed variation in repeatedly measured infant fecal gut microbiota composition and clinically important variables evaluated during the course of this study. Permutational multivariate analysis of variance (PERMANOVA^56^; *Adonis* in R) was performed to determine factors that significantly (*p* < 0.05) explained variation in microbiota *β* diversity at specific time points.

A three model approach (Poisson, negative binomial, and zero-inflated negative binomial mixed-effects models) corrected for multiple testing using the Benjamini–Hochberg method^57^ (*q* < 0.10) as previously described^58^, was used to determine specific Operational Taxonomic Units (OTUs; present in at least 20% of samples) which differed in relative abundance between groups at each time point.

Persistent founder taxa were identified in a subset of infants (*n* = 23) who had five samples (collected at birth, 1, 3, 6, and 12 months) available for analysis. An OTU had to be present, i.e., detected, in meconium, and in all four subsequent samples to be classified as a persistent founder taxon. Taxa that were only present in one infant were excluded from analysis. Differences in the frequency of founder taxa (presence or absence) between HC and HR infants was then assessed using Fisher’s exact test.

Bray Curtis distance was calculated for fecal metabolites in QIIME to assess compositional dissimilarity between samples. Metabolites exhibiting significantly (*p* ≤ 0.05 and *q* ≤ 0.3) different concentrations (log-transformed) between HC vs HRP and HRP vs HRLGG at each time point were identified using two-tailed Welch’s *t*-test and visualized using Cytoscape version 3.4.0^59^. For data generated using the DC/T-cell assay, LME (R package lmerTest) adjusted for blood donor as a random effect was used to test for T cell and cytokine differences. Points plotted reflect the adjusted T-cell and cytokine values.

### Data availability

All sequence data related to this study are available from the European Nucleotide Archive (ENA) under accession number PRJEB20766.

## Electronic supplementary material

Supplementary Information

Description of Additional Supplementary Files

Supplementary Data 1

Supplementary Data 2

Supplementary Data 3

Supplementary Data 4

Supplementary Data 5

Supplementary Data 6

Supplementary Data 7

Supplementary Data 8

Supplementary Data 9
