# Supplementary material for: Delayed gut microbiota development in high-risk for asthma infants is temporarily modifiable by Lactobacillus supplementation
Source: Nat Commun. 2018 Feb 16;9:707. doi: 10.1038/s41467-018-03157-4 (PMC5816017; doi:10.1038/s41467-018-03157-4)
Supplement: Supplementary file 1 — Supplementary Information [file 41467_2018_3157_MOESM1_ESM.pdf]

Supplementary Material:

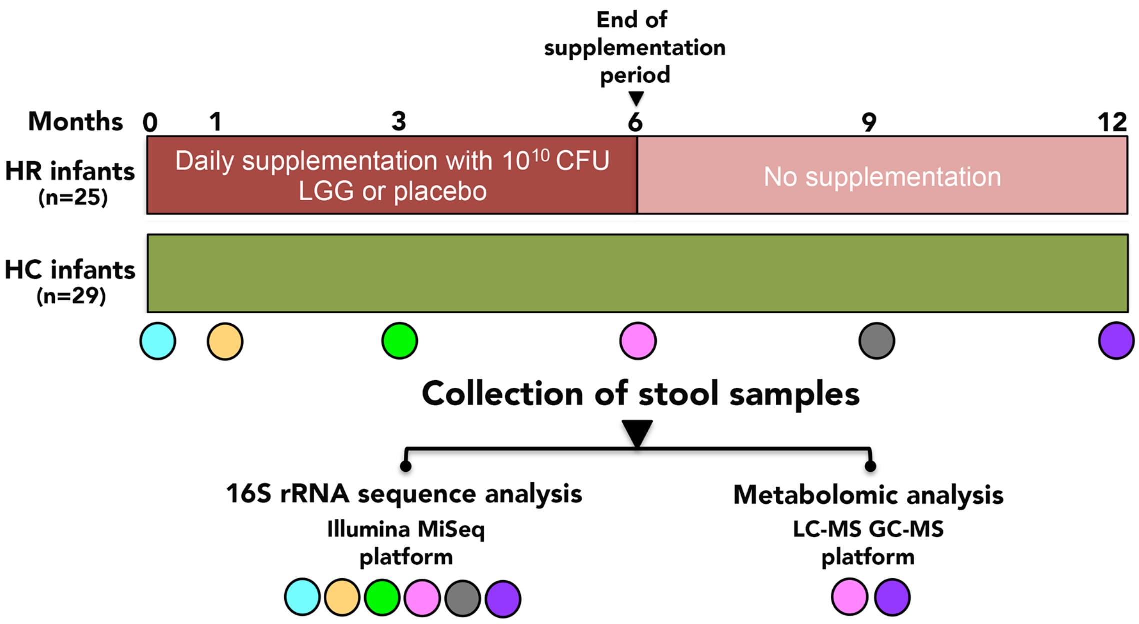

Supplementary Figure 1 Study schema Infants at high risk for asthma (HR) received daily oral supplementation with *Lactobacillus rhamnosus* (LGG) or placebo from birth to age 6 months. Healthy infants (HC) were included as a control group and received no intervention. Stool samples were collected at regular intervals during the first year of life: at birth (meconium, collected within 48 h after delivery), during the supplementation period (at 1, 3, and 6 months) as well as post-supplementation (at 9 and 12 months). All samples (n=253) were analyzed for bacterial microbiota using 16S rRNA sequencing. Metabolic profiles from a subset of paired samples (selected at random) collected at 6 months (n=33) and at 12 months (n=33) were assessed by Liquid Chromatography-Mass Spectrometry (LC-MS) and Gas Chromatography-Mass Spectrometry (GC-MS).

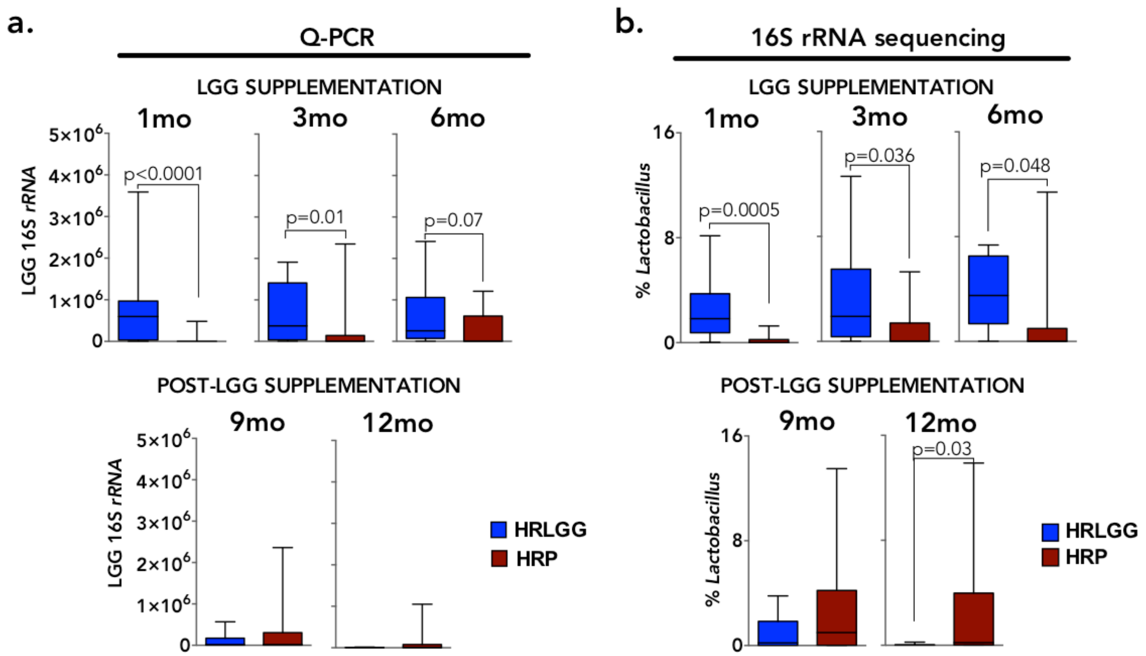

Supplementary Figure 2 High-risk for asthma infants (HR) supplemented with *Lactobacillus rhamnosus* (HRLGG) exhibit significantly greater abundance of *Lactobacillus* compared to HR placebo (HRP) controls during the oral supplementation period a. Abundance of *L. rhamnosus* GG (LGG) quantified by qPCR is significantly higher in HRLGG (n=10) compared with HRP (n=15) infants during the supplementation period (1, 3 and 6 month samples), but not in the period following cessation of supplementation (9 and 12 month samples; Mann-Whitney t-test). **b.** Relative abundance of the genus *Lactobacillus* as assessed by 16S rRNA sequencing independently validates qPCR observations (Mann-Whitney t-test). Whiskers extend to 95% confidence interval.

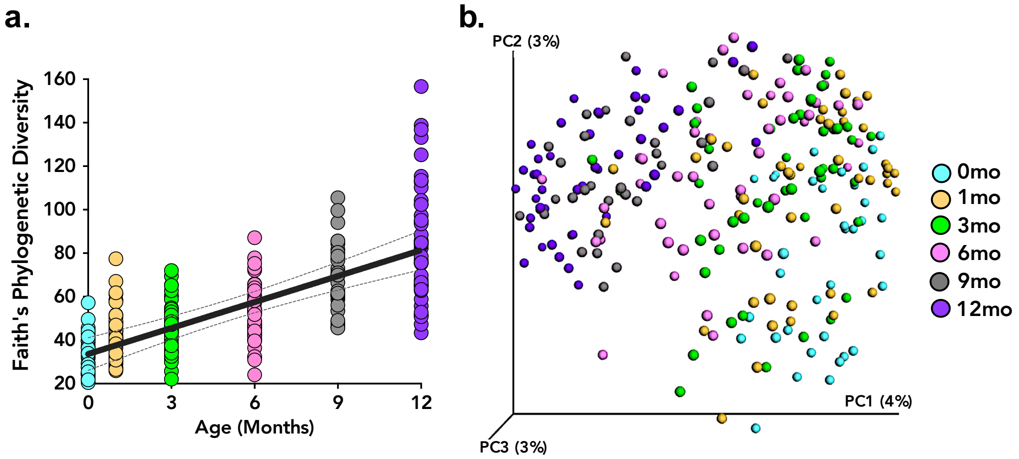

Supplementary Figure 3 Bacterial gut microbiota diversifies and exhibits taxonomic succession with increasing age a. Infant age and Faith's phylogenetic diversity exhibit a positive correlation over the first year of life (Linear Mixed Effects Model, β=4.0±0.4, p<0.0001). **b.** Taxonomic variance in the developing gut microbiota is significantly related to infant age (Unweighted UniFrac distance; Linear Mixed Effects Model; p<2x10^{-16}).

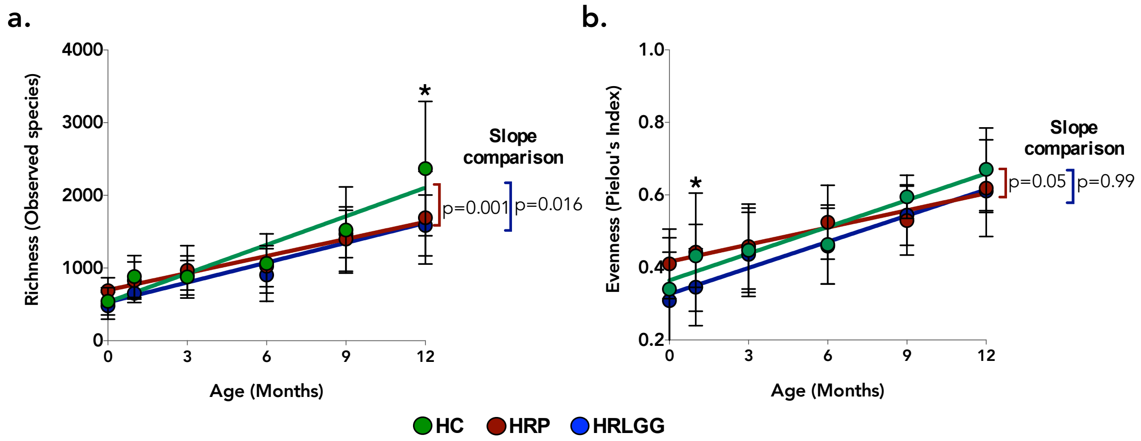

Supplementary Figure 4 Reduced rates of alpha diversification observed in high-risk for asthma (HR) subjects, are due to significantly reduced rates of gain in gut microbiota richness and evenness, only the latter is mitigated in *Lactobacillus rhamnosus*-supplemented (HRLGG) subjects The rate of increase in bacterial community **a.** richness and **b.** evenness over the first year of life is significantly decreased in HR (n=25) subjects and only the evenness disparity is partly rescued by LGG supplementation (β ANCOVA). Significant difference in alpha diversity indices based on cross-sectional analysis are indicated by asterisks (Kruskal-Wallis test, $p<0.05$). Error bars indicate standard-deviation from the mean.

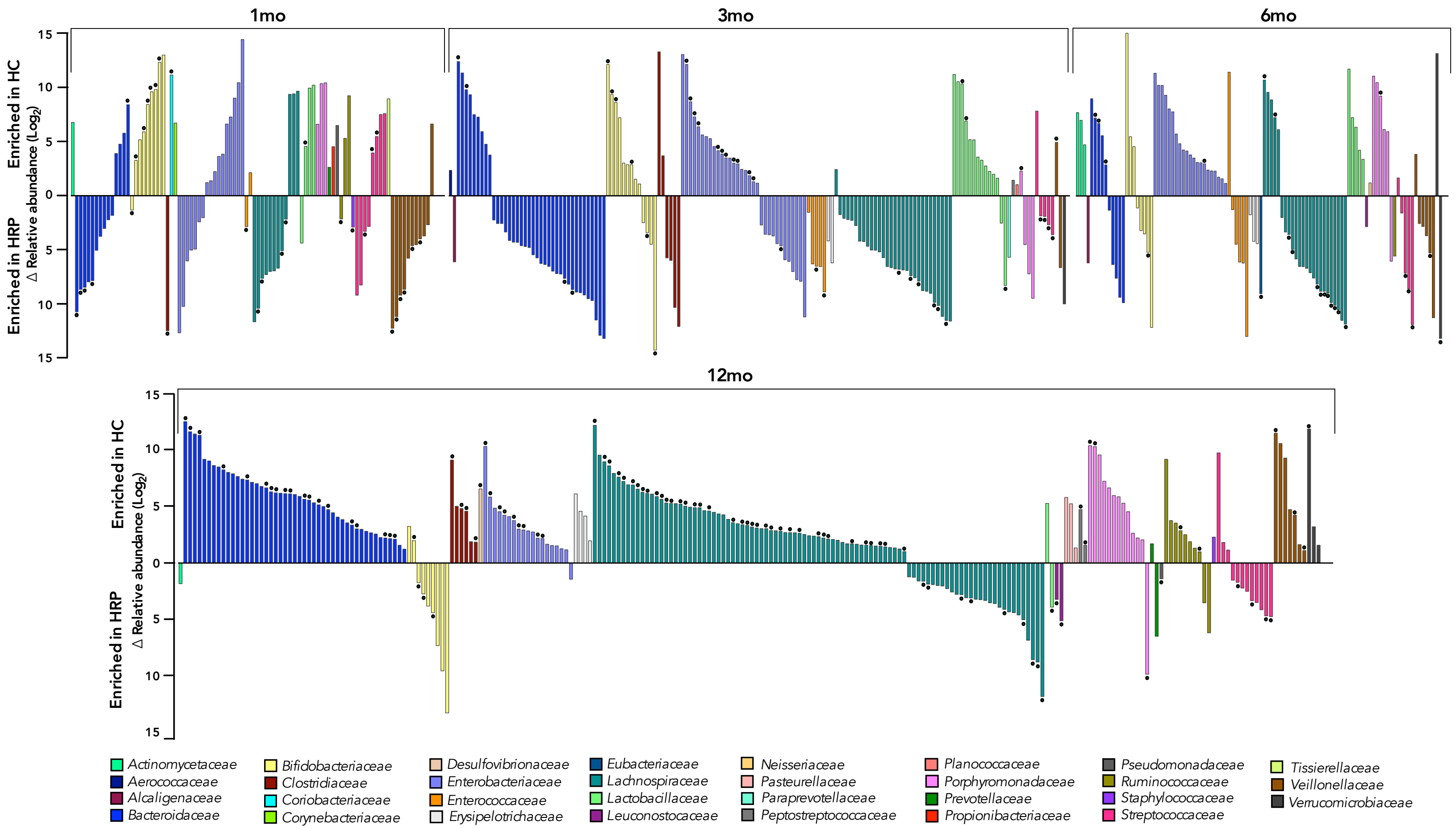

Supplementary Figure 5 Bacterial taxa significantly enriched in healthy (HC) compared to high-risk for asthma placebo-supplemented (HRP) infants at 1, 3, 6 and 12 months of age Significance was determined using best-fit regression model (see methods) and corrected for false discovery, q<0.1. Each bar represents a specific taxon within a bacterial family. Height of bar represents the difference in mean relative abundance. Taxa indicated with a black dot represent those that are also found to discriminate HRLGG infants from HRP, indicating taxonomic overlap between HRLGG and HC subjects that is sustained at 12 months despite cessation of supplementation**.**

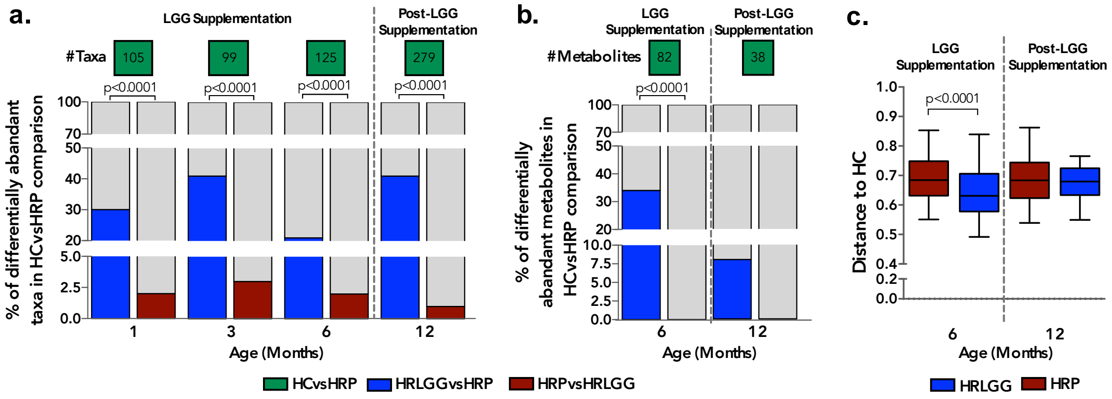

Supplementary Figure 6 Compared with high-risk for asthma (HR) placebo controls (HRP), *Lactobacillus rhamnosus*-supplemented (HRLGG) infants are taxonomically more similar to healthy controls (HC) throughout the study period and exhibit a greater degree of metabolic overlap with HC infants at 6 but not 12 months of age. a. Of the taxa that significantly differ in relative abundance between HC (n=29) and HRP (n=15) subjects (significantly enriched/depleted; Best-fit regression model, corrected for false discovery q<0.1), a significantly greater proportion (Fisher's exact test, p<0.0001) of these specific taxa are also found to discriminate HRLGG (n=10) from HRP subjects (significantly enriched/depleted; Best-fit regression model, corrected for false discovery q<0.1) at all time points examined. **b.** Of the metabolites that significantly differ in abundance between HC (n=15) and HRP (n=11) subjects (Welch's two-sample t-test, p≤0.05), a significantly greater proportion of these specific taxa are also found to discriminate HRLGG (n=7) from HRP subjects at 6 (Fisher's exact test, p<0.0001) but not 12 months of age (Fisher's exact test, p=0.24). **c.** Comparison of beta-diversity distances indicates that compared with HRP (n=11) infants, the metabolic profile of HRLGG (n=7) subjects is marginally more similar to that of HC (n=15) infants at 6 months but not at 12 months of age (Bray Curtis distance, Welch's correction t-test). Whiskers extending to 5th and 95th percentile.

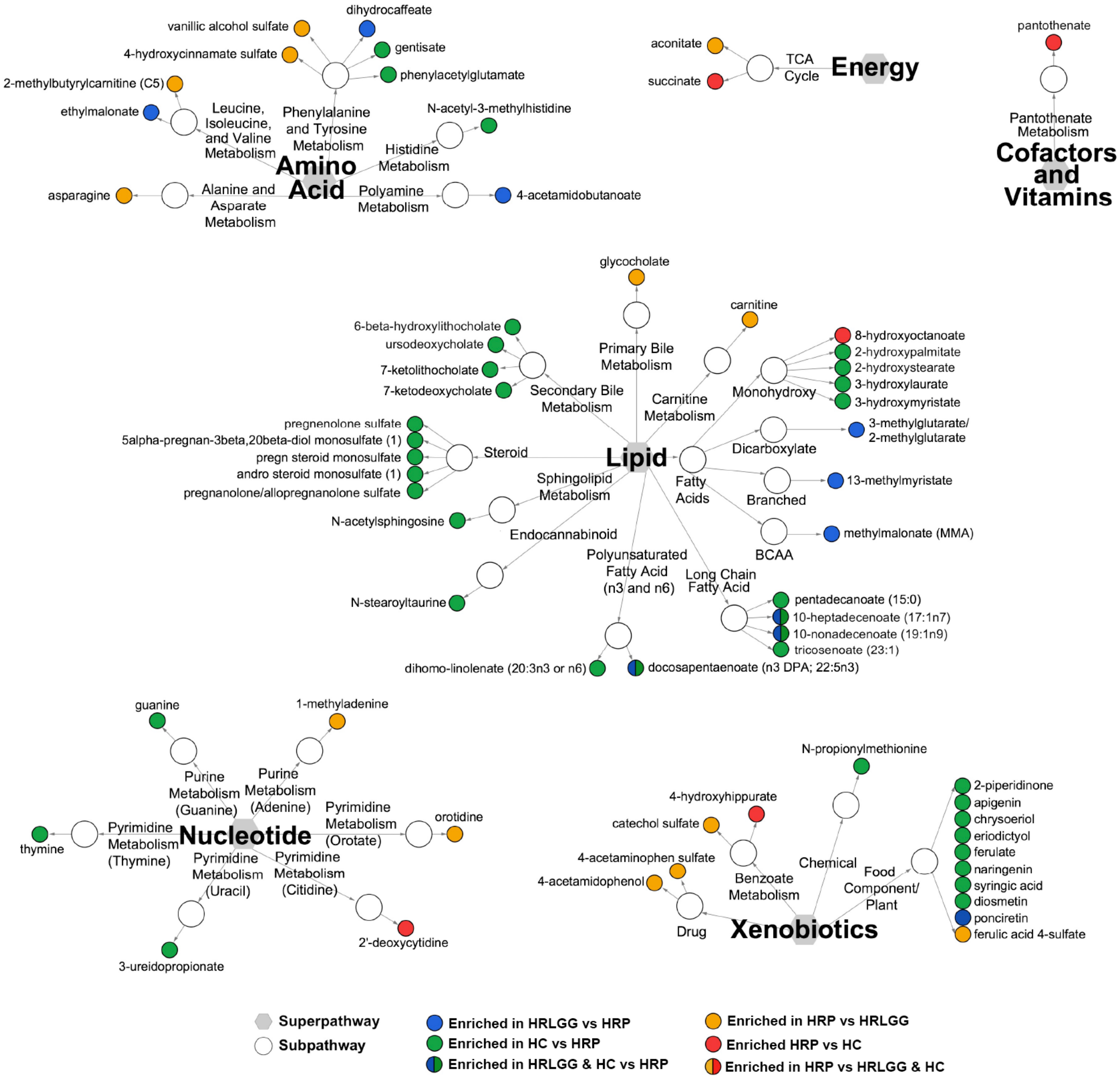

Supplementary Figure 7 Three-way comparison of 12 month fecal samples identifies relatively few discriminatory metabolites enriched in healthy controls (HC; n=15) and *Lactobacillus rhamnosus*-supplemented high-risk for asthma (HRLGG; n=7) subjects compared to high-risk for asthma placebo (HRP; n=11) subjects. Significance was tested using Welch's two-sample *t*-test ($p \leq 0.05$ was considered significant).

Supplementary Table 1 Demographics of high-risk for asthma (HR) and healthy (HC) infants included in this study.

Descriptive variable	HC[&] (n=29)	HR[&] (n=25)	p-value[$]	HRP[&] (n=15)	HRLGG[&] (n=10)	p-value[$]
Mother with history of asthma	**0**/29	**15**/25	**<0.0001**	**10**/15	**5**/10	0.44
Mother with active asthma	**0**/29	**14**/25	**<0.0001**	**10**/15	**4**/10	0.24
Father with history of asthma	**0**/29	**12**/25	**<0.0001**	**5**/15	**7**/10	0.11
Father with active asthma	**0**/29	**9**/25	**<0.001**	**4**/15	**5**/10	0.39
Birth mode (Vaginal)	**25**/29	**22**/25	0.32	**14**/15	**8**/10	0.54
Gestational age[A] (weeks)	**39** (38-40)	**39** (38-40)	0.24[B]	**40** (39-41)	**39** (38-39)	0.12[B]
Mother taking probiotics	**6**/26	**5**/25	1.00	**4**/15	**1**/10	0.35
Antibiotics during delivery	**11**/29	**9**/25	1.00	**5**/15	**4**/10	1.00
Infant treated with antibiotics	**1**/29	**2**/25	0.59	**2**/15	**0**/10	0.50
Exposure to indoor pets	**16**/27	**6**/25	**0.01**	**5**/15	**1**/10	0.35
Breastfeeding duration[A] (months)	**10** (3-12)	**12** (3-12)	0.45[B]	**12** (1-12)	**12** (5-12)	0.71[B]
Eczema diagnosis at 12mo	**2**/27	**7**/25	*0.07*	**5**/15	**2**/10	0.66
Wheeze at 12mo	**6**/27	**8**/25	0.55	**4**/15	**4**/10	0.41
Mode of nutrition						
1mo						
Exclusive breast milk	**19**/27	**15**/20		**9**/12	**6**/8	
Combination	**4**/27	**3**/20		**2**/12	**1**/8	
Exclusive formula	**4**/27	**2**/20	1.00	**1**/12	**1**/8	1.00
3mo						
Exclusive breast milk	**19**/24	**17**/24		**9**/14	**8**/10	
Combination	**2**/24	**4**/24		**3**/14	**1**/10	
Exclusive formula	**3**/24	**3**/24	0.89	**2**/14	**1**/10	0.82
6mo						
Exclusive breast milk	**10**/20	**15**/25		**9**/15	**6**/10	
Combination	**6**/20	**5**/25		**2**/15	**3**/10	
Exclusive formula	**4**/20	**5**/25	0.79	**4**/15	**1**/10	0.47
9mo						
Exclusive breast milk	**4**/9	**13**/24		**8**/14	**5**/10	
Combination	**1**/9	**3**/24		**2**/14	**1**/10	
Exclusive formula	**4**/9	**8**/24	0.86	**4**/14	**4**/10	0.85
12mo						
Exclusive breast milk	**5**/20	**14**/24		**8**/14	**6**/10	
Combination	**5**/20	**1**/24		**1**/14	**0**/10	
Exclusive formula	**7**/20	**8**/24		**4**/14	**4**/10	
Exclusive solids	**3**/20	**1**/24	**0.05**	**1**/14	**0**/10	1.00
In the 1st year of life [#]						
Exclusive breast milk	**13**/28	**13**/25		**8**/15	**5**/10	
Combination	**9**/28	**7**/25		**3**/15	**4**/10	
Exclusive formula	**6**/28	**5**/25	0.94	**4**/15	**1**/10	0.42
Introduction of solid foods						
Eating Solids 6mo	**18**/20	**21**/25	0.68	**13**/15	**8**/10	1.00
Eating Solids 9mo	**9**/9	**24**/24	NA	**14**/14	**10**/10	NA
Eating Solids 12mo	**21**/21	**24**/24	NA	**14**/14	**10**/10	NA

[&]Number of infants/Total number of samples. HRP- HR placebo controls and HRLGG- *Lactobacillus rhamnosus*-supplemented HR infants. [$]Statistical significance was determined using Fisher's exact test. [#]Long-term dietary classification of each infant adjusted for grams/day of formula intake based on reported feeding at collection in the first year of life. [A]Median duration with IQ range. [B]Statistical significance was determined using Mann Whitney test.

Supplementary Table 2 Variables tested in a linear mixed effects model to determine those factors that explain the variability in microbiome composition observed over the first year of life in all participants.

Variable	[a]β (Regression Coefficient)	p-value
Age[#]	-0.029	**< 2×10^{-16}**
Long-term Feeding (Exclusive Formula)[€&]	-0.102	**2.4×10^{-6}**
Duration of breast feeding (months)[&]	0.006	**0.0005**
Mother with active asthma (yes)[&]	0.033	0.105
Mother with history of asthma (yes)[&]	0.029	0.149
Antibiotics during delivery[&]	0.027	0.149
Mode of delivery (C-section)[&]	0.031	0.253
Mother supplemented with probiotics (yes)[&]	0.025	0.263
Long-term Feeding (Combination)[€&]	-0.015	0.394
Father with active asthma (yes)[&]	-0.020	0.403
Father with history of asthma (yes)[&]	-0.018	0.406
Infant treated with antibiotics[&]	0.031	0.421
Supplementation (LGG)[&$]	0.012	0.612
Gestational age (weeks)[&]	-0.003	0.619
Study group (HC vs. HR)[&]	-0.006	0.744
Gender (Male)[&]	-0.005	0.768
Exposure to indoor pets (yes)[&]	0.003	0.850
Supplementation (Placebo)[&$]	0.002	0.938

[a] Regression coefficient values for each variable indicate the degree of unit change in Unweighted UniFrac PCoA1 (and in what direction) occurs in community composition per unit change in the variable. [#]Adjusted for risk of asthma development. [&]Adjusted for age. [$]Compared to HC infants. [€]An overall long-term dietary classification of each infant adjusted for grams/day of formula intake when compared to exclusively breast-fed infants; where combination refers to combination of breast milk and formula.

Supplementary Table 3 Variables tested using PERMANOVA to identify factors that explain meconium microbiota variance.

	**All infants** (n=35)		**Vaginally born infants** (n=31)	
**Variable**	R^2	**p-value**	R^2	**p-value**
Mode of delivery	0.053	**0.001**	-	-
Study group (HC/HR)	0.062	**0.001**	0.060	**0.001**
Parent diagnosed with asthma (mother/father/both)	0.121	**0.002**	0.129	**0.002**
Parent with current active asthma (yes/no)	0.090	**0.002**	0.092	**0.002**
Parent diagnosed with asthma and their current disease status	0.179	**0.004**	0.194	**0.005**
Asthmatic mother with active disease	0.043	**0.019**	0.046	**0.007**
Asthmatic father (yes/no)	0.040	**0.025**	0.044	**0.035**
Asthmatic mother (yes/no)	0.041	**0.027**	0.043	**0.033**
Asthmatic father with active disease	0.037	**0.045**	0.037	0.176
Gestational age (weeks)	0.034	0.130	0.034	0.460
Antibiotics during delivery	0.031	0.346	0.035	0.365
Infant treated with antibiotics	0.029	0.416	0.033	0.508
Indoor pets (yes/no)	0.031	0.420	0.034	0.424
Gender	0.029	0.453	0.030	0.797
Mother taking probiotics (survey at 1mo)	0.026	0.906	0.030	0.705
