# Supplementary material for: Delayed gut microbiota development in high-risk for asthma infants is temporarily modifiable by Lactobacillus supplementation
Source: Nat Commun. 2018 Feb 16;9:707. doi: 10.1038/s41467-018-03157-4 (PMC5816017; doi:10.1038/s41467-018-03157-4)
Supplement: Supplementary file 2 — Description of Additional Supplementary Files [file 41467_2018_3157_MOESM2_ESM.pdf]

## Description of Additional Supplementary Files

File Name: Supplementary Data 1
Description: Specific bacterial taxa relatively enriched or depleted in high-risk for asthma (HR) compared to healthy (HC) infants at birth.

File Name: Supplementary Data 2
Description: Specific bacterial taxa relatively enriched or depleted in high-risk for asthma placebo supplemented (HRP) infants compared to healthy (HC) controls at 1 month of age.

File Name: Supplementary Data 3
Description: Specific bacterial taxa relatively enriched or depleted in high-risk for asthma placebo supplemented (HRP) infants compared to healthy (HC) controls at 3 months of age.

File Name: Supplementary Data 4
Description: Specific bacterial taxa relatively enriched or depleted in high-risk for asthma placebo supplemented (HRP) infants compared to healthy (HC) controls at 6 months of age.

File Name: Supplementary Data 5
Description: Specific bacterial taxa relatively enriched or depleted in high-risk for asthma placebo supplemented (HRP) infants compared to healthy (HC) controls at 12 months of age.

File Name: Supplementary Data 6
Description: Specific bacterial taxa relatively enriched or depleted in high-risk for asthma LGG-supplemented (HRLGG) infants compared to high-risk for asthma placebo (HRP) controls at 1 month of age.

File Name: Supplementary Data 7
Description: Specific bacterial taxa relatively enriched or depleted in high-risk for asthma LGG-supplemented (HRLGG) infants compared to high-risk for asthma placebo (HRP) controls at 3 months of age.

File Name: Supplementary Data 8
Description: Specific bacterial taxa relatively enriched or depleted in high-risk for asthma LGG-supplemented (HRLGG) infants compared to high-risk for asthma placebo (HRP) controls at 6 months of age.

File Name: Supplementary Data 9
Description: Specific bacterial taxa relatively enriched or depleted in high-risk for asthma LGG-supplemented (HRLGG) infants compared to high-risk for asthma placebo (HRP) controls at 12 months of age.
